# Application of Antimicrobial Photodynamic Therapy for Inactivation of *Acinetobacter baumannii* Biofilms

**DOI:** 10.3390/ijms24010722

**Published:** 2022-12-31

**Authors:** Irina Buchovec, Enrika Vyčaitė, Kazimieras Badokas, Edita Sužiedelienė, Saulius Bagdonas

**Affiliations:** 1 Institute of Photonics and Nanotechnology, Faculty of Physics, Vilnius University, Sauletekio av. 3, LT-10222 Vilnius, Lithuania; 2 Institute of Biosciences, Life Sciences Center, Vilnius University, Sauletekio av. 7, LT-10257 Vilnius, Lithuania; 3 Laser Research Center, Faculty of Physics, Vilnius University, Sauletekio av. 10, LT-10223 Vilnius, Lithuania

**Keywords:** *Acinetobacter baumannii*, biofilms, photodynamic therapy, intracellular ROS, pre-incubation, riboflavin, chlorophyllin

## Abstract

*Acinetobacter baumannii* is a dangerous hospital pathogen primarily due to its ability to form biofilms on different abiotic and biotic surfaces. The present study investigated the effect of riboflavin- and chlorophyllin-based antimicrobial photodynamic therapy, performed with near-ultraviolet or blue light on the viability of bacterial cells in biofilms and their structural stability, also determining the extent of photoinduced generation of intracellular reactive oxygen species as well as the ability of *A. baumannii* to form biofilms after the treatment. The efficacy of antimicrobial photodynamic therapy was compared with that of light alone and the role of the photosensitizer type on the photosensitization mechanism was demonstrated. We found that the antibacterial effect of riboflavin-based antimicrobial photodynamic therapy depends on the ability of photoactivated riboflavin to generate intracellular reactive oxygen species but does not depend on the concentration of riboflavin and pre-incubation time before irradiation. Moreover, our results suggest a clear interconnection between the inactivation efficiency of chlorophyllin-based antimicrobial photodynamic therapy and the sensitivity of *A. baumannii* biofilms to used light. In summary, all the analyzed results suggest that riboflavin-based antimicrobial photodynamic therapy and chlorophyllin-based antimicrobial photodynamic therapy have the potential to be applied as an antibacterial treatment against *A. baumannii* biofilms or as a preventive measure against biofilm formation.

## 1. Introduction

Bacterial resistance to antibiotics is one of the most important medical and scientific problems of our time [1,2]. A significant threat comes from the pathogens of the clinically important ESKAPE group (*Enterococcus faecium*, *Staphylococcus aureus*, *Klebsiella pneumonia*, *Acinetobacter baumannii*, *Pseudomonas aeruginosa* and *Enterobacter* spp.), mostly represented by nosocomial infection agents, which are associated with the highest risk of mortality and increased healthcare costs [3]. 

One of the most problematic infection agents of this group is the Gram-negative opportunistic pathogen *A. baumannii*, accounting for about 2% of all nosocomial infections in the USA and Europe [4]. The *A. baumannii*-associated infections are prevalent in intensive care units and are characterized by hospital outbreaks [5]. Immune-compromised and injured patients are the most susceptible to *A. baumannii* infections, which are difficult to treat due to the resistance of *A. baumannii* to most antibiotic classes [6]. Multidrug-resistant (MDR) and extensively drug-resistant (XDR) *A. baumannii* clones have spread in clinical settings worldwide, thereby limiting antibiotic treatment and raising urgent need for new antimicrobials and treatment strategies [7].

*A. baumannii* persistence and spread in the hospital environment are largely attributed to its ability to form biofilms on biotic (human skin, mucous membranes) and abiotic (plastic, glass, metal) surfaces [6]. Biofilms are communities of bacterial cells attached to the surface and surrounded by self-produced extracellular exopolysaccharide matrix [8]. Biofilms contribute to protecting bacteria from antibacterial drugs, harsh environmental conditions, and host immune defense [4]. 

Antimicrobial photodynamic therapy (aPDT) against bacterial biofilms has gained increased attention recently as an alternative strategy to inactivate bacterial pathogens [9]. aPDT is based on the interaction of non-toxic photosensitizer (PS), molecular oxygen, and light of suitable wavelengths to match the PS absorption. After photoexcitation, the PS interacts with molecular oxygen and generates reactive oxygen species (ROS) such as superoxide, hydrogen peroxide, hydroxyl radicals, and reactive singlet oxygen (^1^O_2_) that initiate cellular damage, thereby destroying the bacterial cells [10,11]. The aPDT aims at multiple biofilm targets, such as proteins, lipids, DNA, and extracellular polymeric substance (EPS). ROS generated upon the photoactivation of the PS attack adjacent targets present within the biofilm matrix, inside and outside the bacterial cells [8,9].

aPDT has several advantages over antibiotics, including the ability to inactivate multiple cellular targets, and the lack of resistance to the treatment [12]. The efficiency of aPDT applied for inactivation of biofilms depends on the numerous factors but especially on physicochemical properties of the PS used, the type of bacteria, the matrix composition, and also on the irradiation conditions [9,13]. 

In this study, we aimed to investigate the efficiency of aPDT based on natural photosensitizers, riboflavin- and chlorophyllin, against *A. baumannii* biofilms with the focus on the role of applied irradiation, regimen of incubation with the PS, and the ability of the PS to generate intracellular ROS.

## 2. Results

### 2.1. Inactivation of Planktonic A. baumannii Cells with aPDT

**Figure 1:**
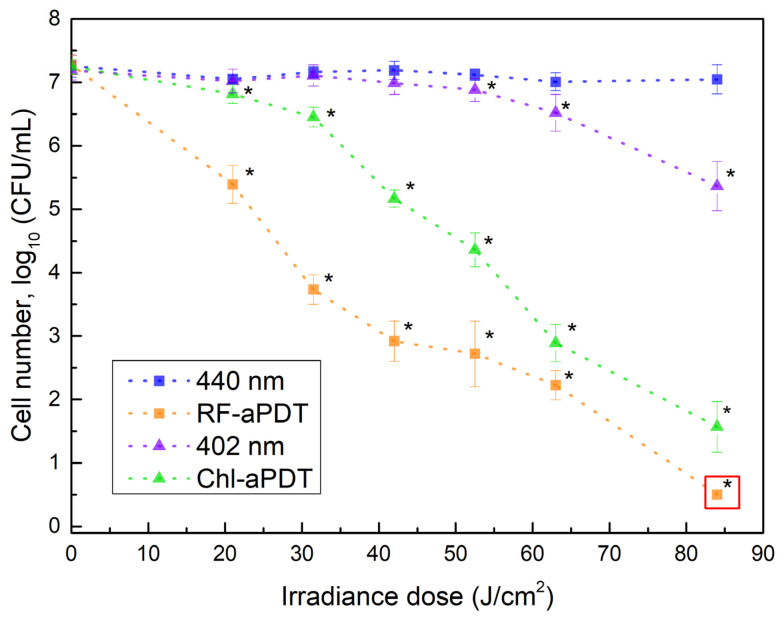
Inactivation of *A. baumannii* planktonic cells by aPDT as a function of irradiance doses (irradiance of 35 mW/cm^2^). Samples in the legend: 440 nm and 402 nm—bacteria in 0.01 M PBS without PS; RF-aPDT—bacteria with 11 µM riboflavin (RF); Chl-aPDT—bacteria with 15 µM chlorophyllin (Chl). Every point is the average of 3–6 independent experiments (10 µL), error bars indicate standard deviation. * indicates statistical significance compared to control culture, *p* < 0.05. The red square means the point detection limit (200 µL 0.5 log_10_). The detection limit of other points is 2 log_10_.

We first evaluated the sensitivity of *A. baumanni* planktonic cells to aPDT and to light alone. The effect of LED light with a peak at 402 nm or 440 nm on the viability of planktonic cells was assessed at 35 mW/cm^2^ irradiance. The irradiance doses ranged from 21 to 84 J/cm^2^ (Table 1). The effect of 440 nm blue light on *A. baumannii* viability was negligible in the whole dose range applied (Figure 1), whereas exposure to 402 nm near-UV light at doses higher than 50 J/cm^2^ progressively decreased *A. baumannii* counts yielding reduction by 1.8 log_10_ after applied 84 J/cm^2^ dose.

aPTD treatment using riboflavin (RF) and chlorophyllin (Chl) dyes efficiently inactivated *A. baumannii* planktonic cells, as shown in Figure 1. *A. baumannii* was found to be more sensitive to riboflavin-based aPDT (RF-aPDT) than to chlorophyllin-based aPDT (Chl-aPDT), especially in the range of the lower irradiance doses. The application of RF-aPTD and Chl-PTD with a higher, 84 J/cm^2^ irradiance dose resulted in 6.7 log_10_ and 5.7 log_10_ inactivation, respectively (Figure 1). The incubation for 40 min with 15 µM Chl or 11 µM RF showed no dark toxicity on the viability of *A. baumannii*. 

### 2.2. Intracellular ROS Generation

**Figure 2:**
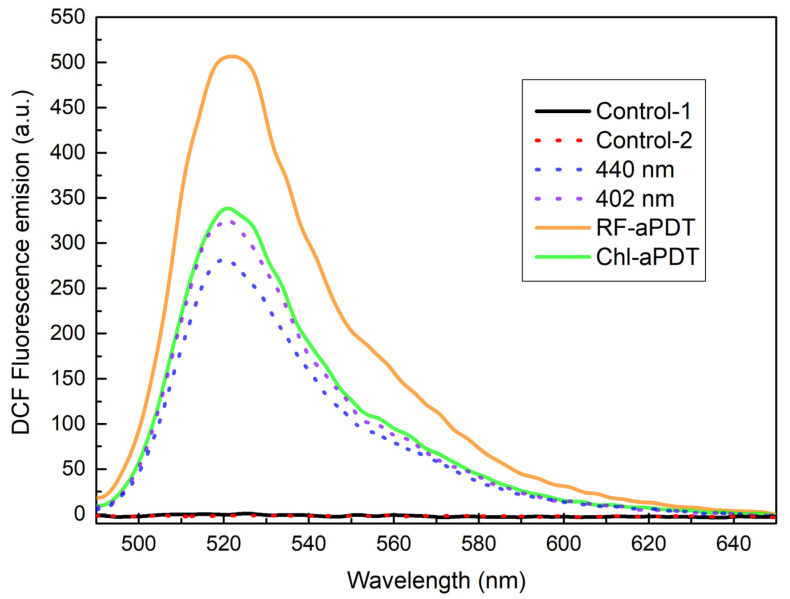
Detection of reactive oxygen species (ROS) activity in *A. baumannii* cells based on measurements of fluorescence spectra of 2′,7′-dichlorofluorescein (DCF). 11 μM RF and 15 μM Chl, irradiance dose of 42 J/cm^2^ for both 440 nm and 402 nm LED light was used. The concentration of bacteria in working solutions was 10^7^ CFU/mL. Samples in the legend: Control-1—*A. baumannii* in 0.01 M PBS buffer without 2′,7′-dichlorofluorescein-diacetate (DCFH-DA); Control-2—*A. baumannii* in 0.01 M PBS buffer with DCFH-DA; 440 nm and 402 nm—Control-2 irradiated with 440 nm and 402 nm, respectively; RF-aPDT—Control-2 + RF + 440 nm irradiation; Chl-aPDT—Control-2 + Chl + 402 nm irradiation.

To compare the pro-oxidant effect of aPDT with irradiation alone and also to ascertain the role of the PS type on the photoinactivation mechanism, we evaluated the ability of Chl and RF PS to generate ROS intracellularly. Intracellular ROS levels were measured in live bacterial cells by using 2′,7′ -dichlorofluorescein-diacetate (DCFH-DA) assay as described in Section 4.5. Generation of ROS was observed as an increase in 2′,7′-dichlorofluorescein (DCF) fluorescence intensity (I_FL_) after irradiation of *A. baumannii* cells in the presence or absence of the PS (Figure 2). RF-aPDT produced the highest peak fluorescence signal, while that in the case of Chl-aPDT was about 1.3-fold lower. On the other hand, exposure of *A. baumannii* samples to 440 nm LED light alone resulted in the lowest DCF signal—I_FL_ at the peak was about 53% of that detected after RF-aPDT. In contrast, the DCF peak fluorescence signal detected after irradiation with 402 nm light alone, reached as much as 91% of the signal measured after Chl-aPDT (Figure 2).

### 2.3. A. baumannii Biofilm Formation after aPDT Treatment 

**Figure 3:**
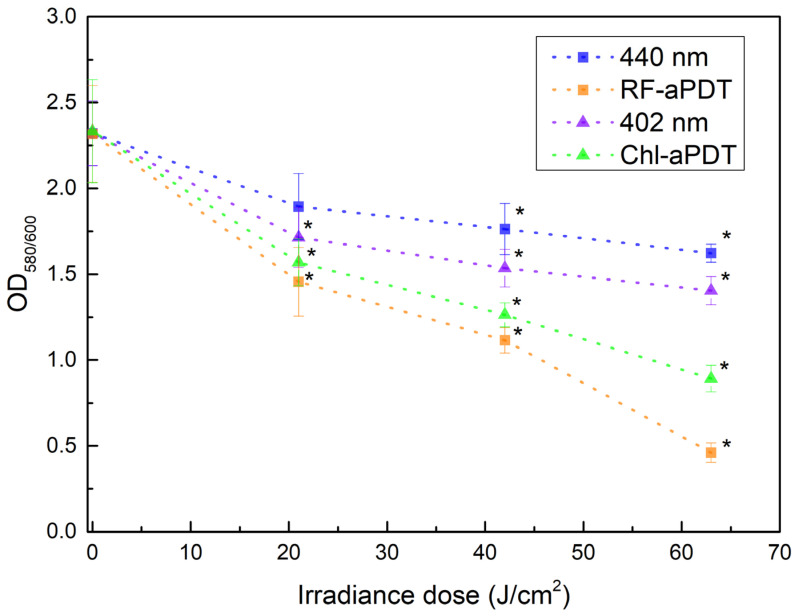
Effect of aPDT on *A. baumannii* biofilm formation as quantified by crystal violet (CV) assay. 440 nm and 402 nm—bacteria irradiated without PS; RF-aPDT—bacteria with 11 µM RF; Chl-aPDT—bacteria with 15 µM Chl. Every point is the average of OD_580/600_ ratio of three independent experiments, error bars indicate standard deviation; * indicates statistical significance compared to control culture, *p* < 0.05.

In order to access the impact of aPTD as a preventive against *A. baumannii* biofilms, the capacity of planktonic cells to form biofilms after RF-aPDT and Chl-aPDT was tested at the beginning. Formed biofilms were quantified using crystal violet (CV) assay as described in Section 4.6. As seen in Figure 3, both exposure to LED light and aPDT decreased the capacity of *A. baumannii* to form biofilms in a dose-dependent manner. Thus, aPDT treatment with irradiance doses ranging from 21 J/cm^2^ to 63 J/cm^2^ reduced the OD_580/600_ value from about 1.6-fold for the lowest dose to 5-fold for the highest dose after RF-aPTD and from 1.5-fold to 2.6-fold after Chl-aPTD (Figure 3). 

The irradiation of *A. baumannii* planktonic culture with LED light alone had less effect on the subsequent formation of biofilms compared to RF-aPDT and Chl-aPDT, albeit it reduced the OD_580/600_ value by 1.4-fold (in the case of 440 nm) and 1.6-fold (402 nm) at 63 J/cm^2^ irradiance dose, as compared to non-irradiated control samples. 

### 2.4. Inactivation of A. baumannii Biofilms with RF-aPDT and Chl-PDT

The efficacy of aPDT using RF and Chl as sensitizers for the inactivation of *A. baumannii* biofilms was investigated. First, we evaluated the dark toxicity of RF and Chl. Biofilms were incubated in the dark with 11 µM or 110 µM RF and 15 µM or 150 µM Chl at room temperature for different periods (Table S1). Higher concentrations of the PS were included since bacterial biofilms are known to be more resistant than planktonic cells to antimicrobial treatments [9,14]. After incubation, bacterial CFUs were determined upon detachment of the biofilm cells as described in the Section 4.7. It has been found that incubation with the PS even at 10-fold higher concentration alone had no impact on *A. baumannii* biofilms, indicating that the PS was nontoxic without irradiation (Table S1).

**Figure 4:**
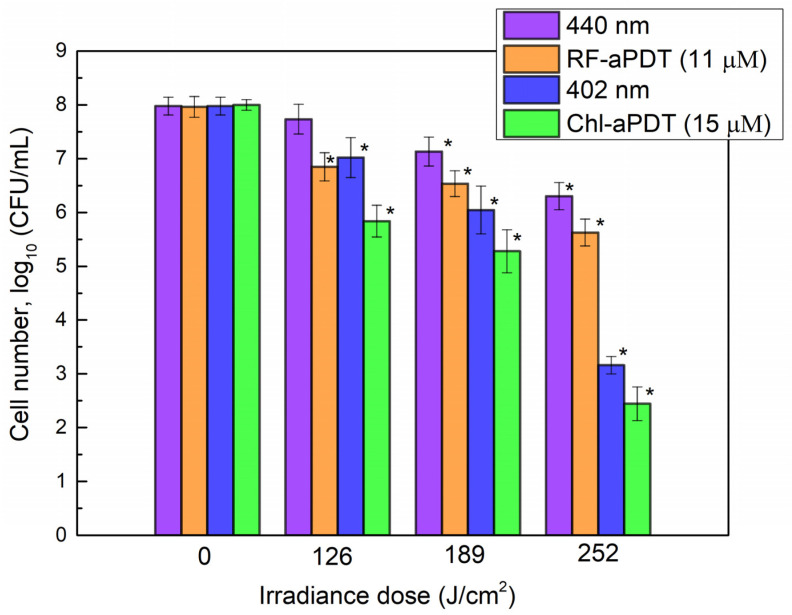
Photodynamic inactivation of *A. baumannii* biofilms by RF-aPDT and Chl-aPDT with 11 μM RF and 15 μM Chl, presented in numbers of the surviving cells as a function of irradiance doses. CFU values present the average of three independent experiments, error bars indicate standard deviation. * Shows statistical significance compared to the control culture, *p* < 0.05.

Next, photoinactivation of *A. baumannii* biofilms after exposure to 402 nm and 440 nm LED light alone or after Chl-aPDT and RF-aPDT treatment was investigated by applying higher irradiance doses (Figure 4). The viability of *A. baumannii* biofilm cells decreased by 0.9 log_10_ and 1.9 log_10_ after exposure to 126 J/cm^2^ and to 189 J/cm^2^ doses of 402 nm light, respectively. The highest applied irradiance dose of 252 J/cm^2^ resulted in a viability loss by 4.8 log_10_. The effect of irradiation with 440 nm light was less pronounced and yielded reduction in CFUs by 1.7 log_10_ at the highest 252 J/cm^2^ dose applied (Figure 4).

The combination of light and 11 µM RF or 15 µM Chl had a modest bactericidal effect on the biofilm cells compared to the light alone and resulted in a CFU reduction by 5.6 log_10_ and 2.3 log_10_, respectively, at 252 J/cm^2^ dose.

**Figure 5:**
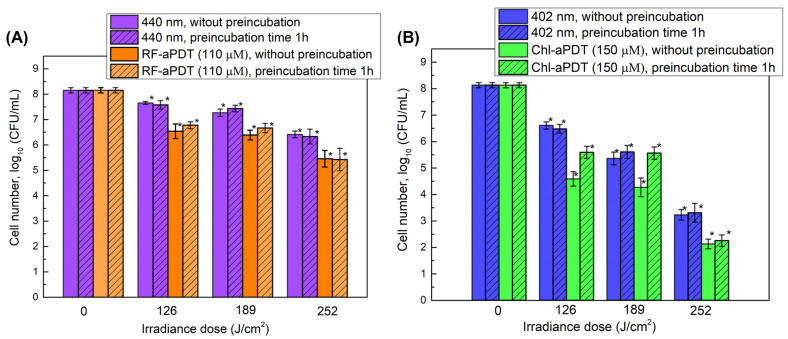
Photodynamic inactivation of *A. baumannii* biofilms by aPDT with 110 μM RF (**A**) or 150 μM Chl (**B**) without preincubation and with 60 min preincubation in the dark at room temperature followed by corresponding irradiation (irradiance doses shown in Table 1). The effect of light alone was determined for bacterial samples without PS at the same conditions. CFU values present the average of three experiments, error bars indicate standard deviation. * shows statistical significance compared to the control culture, *p* < 0.05.

To improve the efficacy of aPDT treatment on *A. baumannii* biofilms, we used the higher concentrations of PSs and added a step of preincubation with the PS to the aPDT regimen. It has been reported that preincubation of bacterial biofilms with the PS before irradiation can increase the efficiency of aPDT, as more PS can penetrate the cells in the biofilms [15,16,17]. In the presented experiments, an increase in the efficiency of aPDT was observed with a higher Chl concentration, but not at the highest irradiation dose used. The *A. baumannii* CFU number decreased by 3.5 log_10_ after the photoactivation of 150 µM Chl with 402 nm light at 126 J/cm^2^ (Figure 5B). As can be seen in Figure 5A, application of a higher concentration of RF (110 μM) had no additional impact on the efficiency of RF-aPDT against the biofilms as compared to 11 μM RF-aPDT (Figure 4), and reduction in CFU of a similar range was observed at all irradiance doses used. The preincubation step using 110 μM RF had no impact on the efficiency of RF-aPDT compared to the non-preincubated samples, whereas in the case of Chl-aPDT it resulted in a reduced CFU loss as compared to the non-preincubated biofilm samples (Figure 5B).

### 2.5. Quantification of Biofilms after aPDT 

**Figure 6:**
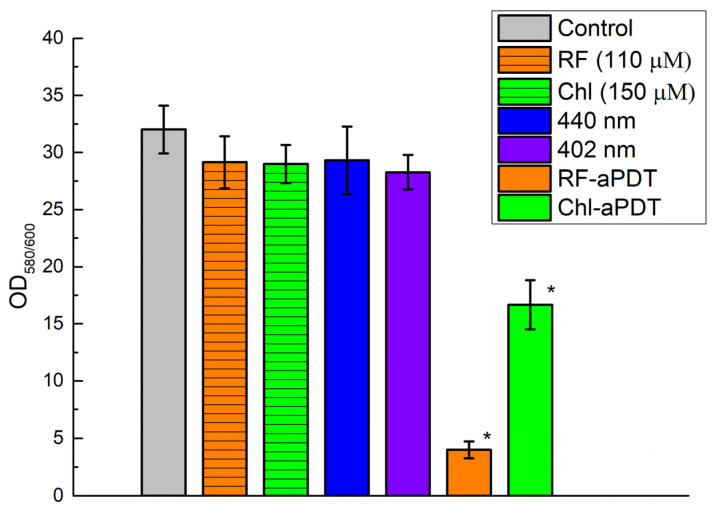
Effect of aPDT on the destruction of *A. baumannii* biofilms as quantified by crystal violet (CV) assay. Samples: Control—without PS and non-irradiated; 440 nm and 402 nm—irradiated without PS; RF-aPDT—with 110 µM RF and irradiated with 440 nm; Chl-aPDT—with 150 µM Chl and irradiated with 402 nm. Every point is the average of OD_580/600_ ratio of three independent experiments, error bars indicate standard deviation, * indicates statistical significance compared to control culture, *p* < 0.05.

To quantify the remaining *A. baumannii* biofilms after Chl-aPDT and RF-aPDT treatment using 110 μM RF or 150 μM Chl and exposing to 126 J/cm^2^, CV assay was applied as described in Section 4.6. As shown in Figure 6, for the chosen combinations RF-aPDT had a higher anti-biofilm effect than Chl-aPDT resulting in an 8-fold reduction in the OD_580/600_ value compared to a 1.9-fold reduction in the latter case. The results also demonstrated no significant difference in the damaging effect on the biofilms exposed with 402 nm and 440 nm LED light alone or after 1 h incubation PS in the dark compared to the control samples (Figure 6).

## 3. Discussion

Bacterial biofilms cause up to 80% of chronic and recurrent infections, and their resistance to antibiotics is 10–1000 times higher than planktonic cells [14]. Since *A. baumannii* is known for multiple resistance mechanisms and a strong capacity to form biofilms [5], the available options for the pharmacological treatment of infections caused by these pathogens are limited. The efficiency of aPDT is known to strongly depend on the physicochemical properties of the chosen photosensitizer [9]. The PS of different chemical classes (phenothiazinium dyes, xanthene dyes, tetrapyrrolic compounds, fullerenes, and flavins) has been used to inactivate *A. baumannii* [18]. Some studies have demonstrated a good bactericidal effect against *A. baumannii* planktonic cells; however, only a few studies have been focused on biofilms [18]. 

Natural flavonoid RF and tetrapyrrole Chl have also been considered as a potential PS for the antimicrobial treatment of opportunistic pathogens [19,20]. Our previous study showed the susceptibility of planktonic *A. baumannii* to RF-aPDT and Chl- aPDT [21]. In the present study, we show that the enhanced bactericidal effect of aPDT on *A. baumannii* planktonic cells and their reduced capacity to form biofilms could be achieved by increasing the irradiance dose. This is an important observation, implying that the photosensitization of bacterial cells grown in the planktonic population or at the initial phase of attachment to the surface may minimize the likelihood of subsequent formation of the more resistant biofilm population.

Remarkably, the exposure of the *A. baumannii* planktonic population to 402 nm LED light alone showed a stronger bactericidal effect than 440 nm LED light. In general, the sensitivity of *A. baumannii* to the blue or near-UV light can be explained by the fact that bacteria possess endogenous photosensitizing chromophores such as coproporphyrin III, which, after absorbing light, exerts an antibacterial effect by inducing ROS-mediated cytotoxicity in bacterial cells [22]. While it is implied that ROS formed during irradiation (by light alone and in combination with the PS) play a significant role in bacterial inactivation, there is no clear answer that could explain the extent of the antibacterial effect of the blue light. The role of photoreceptors, chemoreceptors, and DNA repair-related genes in eliciting protective pathways against oxidative stress or modulating bacterial physiological characteristics under blue light irradiation could also be important [23].

In this study, we compared the ability of Chl and RF after photoactivation with near-UV and blue LED light (402 nm and 440 nm, respectively) to generate intracellular ROS in *A. baumannii* with the photoinduced ROS generation by light alone. The measurement of intracellular ROS in bacteria was performed using the 2′,7′-dichlorofluorescein-diacetate (DCFH-DA) staining method [24]. This method is usually used to detect the total intracellular ROS, including hydroxyl radicals (•OH), hydrogen peroxide (H_2_O_2_), and peroxyl radicals (ROO•) [25]. The fluorescence signal of the produced DCF is directly proportional to the amount of ROS generated in cells. The highest DCF fluorescence signal was obtained after RF-aPDT, which indicates that RF can enter planktonic *A. baumannii* cells and generate higher amounts of intracellular ROS in irradiated bacteria. It is important to note that the extent of the intracellular ROS generation in *A. baumannii* boosted the antibacterial effect of the photoactivated RF, but not that of 440 nm LED light irradiation alone at 42 J/cm^2^. This could indicate that bacterial cells are capable to cope with ROS generated by light alone and imply the dependence of the damage on the localization of the PS. These results are supported by other studies [26,27,28] demonstrating that the photoactivated RF does produce ROS, which leads to inactivation of planktonic bacteria. Khan et al. [26] demonstrated a significant increase in the DCF fluorescence level in *Escherichia coli* cells treated with 50 µM RF and white light (27.8 J/cm^2^), indicating intracellular ROS formation. Other authors [27] found that 50 µM RF photoactivated with the blue 450 nm light increased the DCF fluorescence intensity up to 16.8-fold in *E. coli* cells compared to the control, which together with the light only group showed no fluorescence increase. Kim et al. [28] demonstrated a similar fluorescence increase in *E. coli* cells when the ROS generation upon treatment with 50 µM RF and blue LED light (30 mJ/cm^2^) was about 16 times higher than in control.

In contrast, the intracellular ROS generation reflected by the DCF fluorescence signal after Chl-aPDT was 1.5-fold lower compared to RF-aPDT and was comparable to that obtained after irradiation with 402 nm LED light only. These results imply that Chl does not penetrate into the bacterial cells, and during Chl-aPDT the cellular damage is mainly caused by externally generated ROS, presumably, the type II reaction resulting in the formation of singlet oxygen. This is consistent with other studies, where negatively charged PS (some porphyrins and Rose Bengal) in suspensions have been applied to effectively inactivate the Gram-negative pathogens such as *Salmonella enterica* [29] or *E. coli* [30,31].

The effect of aPDT on the biofilms strongly depends on the interaction between the PS and the matrix [9]. The PS could be blocked by the matrix EPS and may not reach the cells in the biofilms. Otherwise, some of the photosensitizers pass through the EPS and come into contact with the bacterial cells. Then the PS binds to the cell surfaces or reaches into the cellular cytoplasm to generate ROS after photoexcitation [9,32]. In this context, it seems appropriate to use the preincubation of the PS with bacteria before irradiation [33]. The primary results of the *A. baumannii* biofilm inactivation with RF-aPDT and Chl-aPDT, presented in our previous study [21] were obtained only with the usage of the pre-incubation step and showed that the *A. baumannii* biofilm cells were more susceptible to near-UV light (402 nm LED light) and Chl-aPDT than to blue light (440 nm) and RF-aPDT. In the present study, we compare the antibacterial effects of both treatments with and without pre-incubation before irradiation. The results confirmed that in all those cases Chl-aPDT was more effective against the biofilms than RB-aPDT, presumably, due to the combined activity of light and aPDT, given that A. *baumannii* cells are more sensitive to 402 nm light alone. However, the preincubation period negatively influenced the Chl-aPDT efficiency on *A. baumannii* cells in the biofilm. Such a finding can be explained by the fact that Chl, like most porphyrins, tend to aggregate in aqueous medium [34]. The RF-aPDT showed similar efficacy in reducing the capacity of the viable *A. baumannii* bacterial count in the biofilms in both cases (without and with pre-incubation) before irradiation. Moreover, no significant differences were seen with the increasing RF concentration. When the remaining *A. baumannii* biofilms were quantified after the RF-aPDT treatment, it was found that RF-aPDT resulted in a stronger biofilm destruction than Chl-aPDT, which may indicate a protective role of the biofilm matrix in the preservation of bacterial cells. 

In summary, all the analyzed results suggest that RF-aPDT and Chl-aPDT have the potential to be applied as methods of antibacterial treatment against *A. baumannii* biofilms or as preventive measures against biofilm formation. We found that the antibacterial effect of RF-aPDT with the chosen irradiation depended only on the ability of the photoactivated RF to generate ROS. In the case of Chl-aPDT, the choice of wavelength of light revealed its importance, as it can provide an additional antibacterial effect, most likely, by exciting endogenous chromophores. Moreover, our observations indicate that RF- and Chl-based aPDT anti-biofilm efficiency against *A. baumannii* does not necessarily benefit from the preincubation with a PS; therefore, optimization of standard aPDT treatment schemes, suitable for planktonic cell populations, should be taken into consideration, when applying aPDT treatment for bacterial biofilms.

## 4. Materials and Methods

### 4.1. Preparation of Photosensitizers 

**Figure 7:**
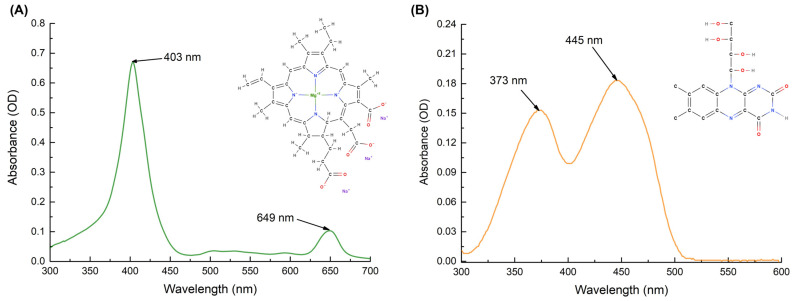
Absorption spectra of 15 µM Chl (**A**) and 11 µM RF (**B**) solutions in distilled water. Chemical structures of PSs are shown.

Non-copperized chlorophyllin sodium salt (Chl) (Carl Roth, Karlsruhe, Germany) (Figure 7A), and riboflavin (RF) (Sigma-Aldrich, St. Louis, MO, USA) (Figure 7B) were used as photosensitizers (PSs). A stock solution of 150 µM Chl (pH 7.2) was prepared by dissolving Chl in distilled water, without any heating or shaking. In contrast, a stock solution of 110 µM RF (pH 6.8) was prepared by stirring (~55 rpm) RF in distilled water at 50 °C for 4 h. The solution was sterilized with a 0.22 µm syringe filter (Carl Roth, Karlsruhe, Germany, ROTILABO^®^ PVDF, P666.1) and stored at 4 °C in the dark before use [21].

The absorption spectra of samples prepared from RF and Chl stock solutions were recorded on a UV/Vis spectrophotometer (Thermo Scientific, GENESYS 10S UV/Vis; Waltham, MA, USA) (Figure 7). All working solutions were freshly prepared by diluting with 0.01 M PBS buffer on the day of use. Polymethyl methacrylate cuvettes of 1 cm were used for the measurements.

### 4.2. Bacterial Strain 

*A. baumannii* clinical isolate II-a [35] was chosen for the aPDT approach study. The bacterial culture was grown at 37 °C in Luria–Bertani (LB) medium (Carl Roth, Karlsruhe, Germany) under constant 144 rpm shaking (GVL, Burgwedel, Germany) overnight (~16 h). Then, the overnight culture was inoculated to a fresh LB media and grown under the same conditions until optical density (OD) at 600 nm (OD_600_) reached a value of 0.65 corresponding to the concentration of 3–4 × 10^8^ colony forming units (CFU)/mL. Bacterial cells were harvested by centrifugation (10 min, 6 °C, 7000× *g*), suspended in 0.01 M PBS (pH 7.4) and immediately used for the aPDT experiments. The concentration of bacteria in working solutions was 10^7^ CFU/mL

### 4.3. Light Source for aPDT

A light emitting diode (LED)-based light source (constructed at the Institute of Photonics and Nanotechnology of Vilnius University) emitted light (a peak intensity at λ = 402 nm or λ = 440 nm) with irradiance 35 mW/cm^2^ at the surface of samples (a distance of 7 cm), which was used for the aPDT experiments [21]. The applied irradiance doses are shown in Table 1.

### 4.4. aPDT of A. baumannii Planktonic Cells

*A. baumannii* was grown as described in Section 4.2. Two pairs of bacterial samples in PBS buffer were prepared, consisting of a control sample with bacteria (Control) and a bacterial sample with PS (15 µM Chl or 11 µM RF). For the photoinactivation, 200 μL of the samples (control and with PSs) was placed into a sterile 96-well microtiter polystyrene plate (MtP) without preincubation period and exposed to the LED light for different time intervals (Table 1). For irradiation, LEDs with peaks of 402 nm and 440 nm were used in Chl-based aPDT and RF-based aPDT, respectively. At the same time, non-irradiated samples of *A. baumannii* (control and with PSs) were incubated in the dark at room temperature. The antibacterial activity of photoactivated Chl and RF using 402 nm and 440 nm LED light on *A. baumannii* viability was evaluated by the microdilution method [36]. Particularly, 10 μL of a diluted bacterial suspension after treatment was inoculated onto the surface of LB agar (LBA) plate (method detection limit 2 log_10_ CFU/mL). If no colonies were detected, a whole volume of treated bacterial suspension (200 μL) was spread afterwards on LBA plates (method detection limit 0.5 log_10_ CFU/mL). LBA plates were kept for 24 h at 37 °C. After incubation, the colonies were counted, an average value was calculated for every point (from 3 to 6 experiments) and bacterial cells counts were recalculated from CFU/mL into log_10_/mL.

### 4.5. Detection of Intracellular ROS 

The measurement of ROS, generated inside *A. baumannii* cells after RF-aPDT and Chl-aPDT, was performed using the 2′,7′-dichlorofluorescein-diacetate (DCFH-DA) method assay as described by Misba et al. [37]. The bacterial strain was grown as described in Section 4.2. Then 10^7^ CFU/mL of *A. baumannii* cells was incubated with 5 μM DCFH-DA (Sigma-Aldrich, St. Louis, MO, USA) for 10 min in PBS (pH 7.4) at 37 °C (Control-2 group). After incubation, 3.6 mL of solution was mixed with either 0.4 mL of PS stock solutions (final concentrations of RF and Chl were 11 µM and 15 µM, respectively, for RF-aPDT and Chl-aPDT groups), or with 0.4 mL of PBS (for 402 nm and 440 nm groups) and irradiated with a dose of 42 J/cm^2^. Then the ROS were quantified by a fluorescence spectroscopy method using a spectrophotometer (Perkin Elmer LS55, Waltham, MA, USA). The excitation (λ_ex)_, emission (λ_em_) wavelengths, and a width of both slits were set as λ_ex_ = 470 nm, λ_em_ = 525 nm and 5 nm, respectively. Polymethyl methacrylate cuvettes of 1 cm were used for the measurements.

### 4.6. A. baumannii Biofilm Formation after aPDT

**Figure 8:**
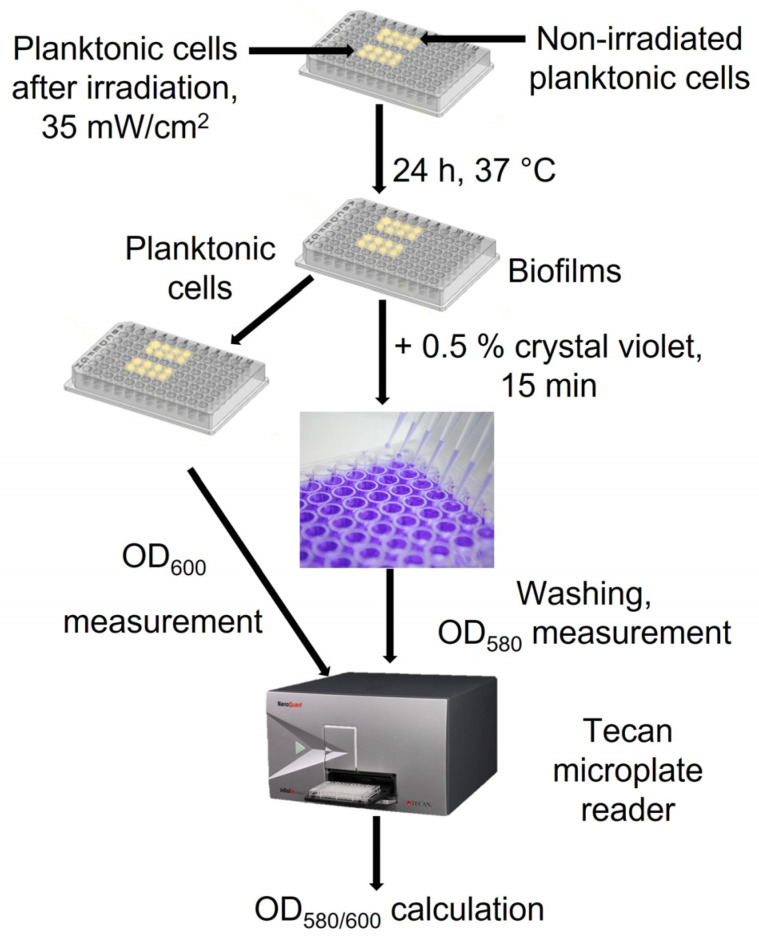
Scheme of *A. baumannii* biofilm formation assay after aPDT.

*A. baumannii* planktonic culture was grown as described in Section 4.2. The irradiation experiments were conducted as described in Section 2.3, and the irradiance doses were 21 J/cm^2^, 42 J/cm^2^ and 63 J/cm^2^. After irradiation, 4 μL of cell suspensions were transferred to a sterile MtP with 196 μL of fresh LB medium and incubated in static conditions for biofilm formation at 37 °C for 24 h (Figure 8). The non-irradiated control samples were also prepared in the same way. The capacity of *A. baumannii* cells to form biofilms was assessed using crystal violet staining according to Skerniškytė et al. [35]. Firstly, OD_600_ of unattached planktonic cells was measured in supernatants taken from the wells by means of a microplate reader (Tecan infinite M200 pro, Tecan, Männedorf, Switzerland). The wells were then washed three times with 0.01 M PBS buffer to remove residual non-adherent bacteria. Biofilms were stained with 0.5% CV dye for 15 min and washed four times with 0.01 M PBS. Dye was eluted with 96% ethanol by incubation for 10 min, and OD_580_ was determined by means of a microplate reader. The biofilm forming capacity was expressed as a OD_580/600_ ratio.

### 4.7. aPDT for Inactivation of A. baumannii Biofilms

*A. baumannii* biofilms were grown in sterile 96-well MtPs (in LB medium) as described in Section 4.6. Biofilms were gently washed with sterile 0.01 M PBS (pH 7.4). Then wells with biofilms were filled with PS solutions (11 µM RF or 15 µM Chl) or with 0.01 M PBS (Figure S1). The biofilms were immediately irradiated with corresponding LED light at 35 mW/cm^2^ After irradiation, biofilms were mechanically detached from the wells and vigorously vortexed. aPDT efficacy was evaluated by determining the CFU on LBA plates, as described in Section 2.4.

In an effort to increase the aPDT efficiency, a set of *A. baumannii* biofilm samples were treated with PS at tenfold higher concentrations (110 µM RF and 150 µM Chl) and incubated in the dark at room temperature (approx. 25 °C) for 60 min. before irradiation (Supplementary Figure S1). Then the samples were exposed to various irradiance doses indicated in Table 1. The biofilms of the non-irradiated controls were detached immediately after preincubation. aPTD efficiency was evaluated by counting CFU as described above.

The effect of aPTD on biofilm integrity was assessed by CV assay according to Skerniškytė et al. [33] with few modifications. After incubation of each suspension (10^7^ CFU/mL) in static conditions for biofilm formation at 37 °C for 24 h, wells with biofilms were exposed to either PS (110 µM RF or 150 µM Chl), LED light (440 nm or 402 nm), or aPDT (RF-aPDT or Chl-aPDT). Then OD_600_ of detached cells was measured, as described in Section 4.6. The wells were gently washed with 0.01 M PBS buffer three times, stained with 0.5% CV dye for 15 min, and washed three to four times with 0.01 M PBS. Next, the dye was eluted with 96% ethanol during incubation for 10 min, and OD_580_ was determined by a microplate reader. The OD_580/600_ ratio was calculated to evaluate the relative level of destruction of biofilm.

### 4.8. Statistical Analysis 

All experiments were repeated 3–6 times. A standard deviation was estimated for every experimental point from values of at least three independent experiments and shown in the figures as an error bar. Data were processed with Origin Pro 9.1 software (OriginLab Corporation, Northampton, MA, USA) and were statistically analyzed using One-way Analysis of Variance (ANOVA). The post hoc Bonferroni test was used for the comparison between the experimental groups and the control group. The level of significance was set at *p* < 0.05.

## Figures and Tables

**Table 1:** Irradiance doses at 402 nm and 440 nm used in experiments.

	Planktonic Cells						Biofilms		
**Time (min)**	10	15	20	25	30	40	60	90	120
**Irradiance dose** **(J/cm^2^)**	21	31.5	42	52.5	63	84	126	189	252

The irradiance dose (J/cm^2^) was calculated as irradiance (mW/cm^2^) multiplied by irradiation time (s).

## Supplementary Materials

The following supporting information can be downloaded at: https://www.mdpi.com/article/10.3390/ijms24010722/s1.
