# Supplementary material for: Application of Antimicrobial Photodynamic Therapy for Inactivation of Acinetobacter baumannii Biofilms
Source: Int J Mol Sci. 2022 Dec 31;24(1):722. doi: 10.3390/ijms24010722 (PMC9820809; doi:10.3390/ijms24010722)
Supplement: Supplementary file 1 [file ijms-24-00722-s001.zip › Figure S1.pdf]

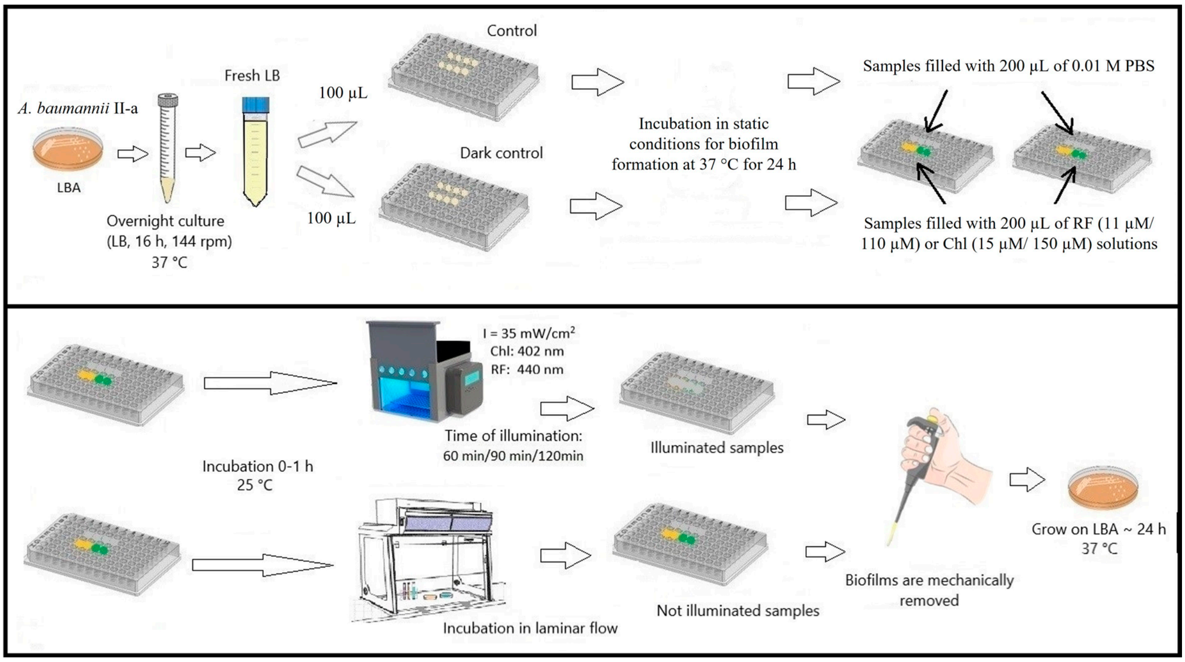

Figure S1. Inactivation of *A. baumannii* biofilm cells. Top—preparation of samples; Bottom—inactivation of cells using aPDT; LB—Luria–Bertani medium; LBA—LB agar plate; Control—bacteria in 0.01 PBS without PS; Dark control—bacteria with PS.
