# Supplementary material for: Application of Antimicrobial Photodynamic Therapy for Inactivation of Acinetobacter baumannii Biofilms
Source: Int J Mol Sci. 2022 Dec 31;24(1):722. doi: 10.3390/ijms24010722 (PMC9820809; doi:10.3390/ijms24010722)
Supplement: Supplementary file 1 [file ijms-24-00722-s001.zip › Table S1.pdf]

Table S1. Dark toxicity effect of RF and Chl on the viability of *A. baumannii* biofilms cells (reported in $log_{10}$ scale).

| **Incubation time, min** | **0** | **60** | **90** | **120** | **150** | **180** |
|---|---|---|---|---|---|---|
| **Control-1*** | 8.05 ± 0.09 | 8.06 ± 0.10 | 8.14 ± 0.09 | 8.17 ± 0.11 | 8.09 ± 0.09 | 8.09 ± 0.12 |
| **RF**** | 8.08 ± 0.09 | 8.05 ± 0.08 | 8.17 ± 0.11 | 8.12 ± 0.14 | 8.19 ± 0.12 | 8.03 ± 0.14 |
| **Chl***** | 8.05 ± 0.08 | 8.16 ± 0.07 | 8.11 ± 0.10 | 8.05 ± 0.09 | 8.10 ± 0.09 | 8.07 ± 0.12 |

** Control-1: bacteria biofilms without PS; ** RF: bacteria with 110 μM of RF; *** Chl: bacteria with 150 μM of Chl;*
